# Trends in liver transplantation for primary biliary cirrhosis in the Netherlands 1988-2008

**DOI:** 10.1186/1471-230X-10-144

**Published:** 2010-12-20

**Authors:** Edith MM Kuiper, Bettina E Hansen, Herold J Metselaar, Robert A de Man, Els B Haagsma, Bart van Hoek, Henk R van Buuren

**Affiliations:** 1 Department of Gastroenterology and Hepatology, Erasmus University Medical Center, Rotterdam, The Netherlands; 2 Department of Epidemiology and Biostatistics, Erasmus University Medical Center, Rotterdam, The Netherlands; 3 Department of Gastroenterology and Hepatology, University Medical Center Groningen, Groningen, The Netherlands; 4 Department of Gastroenterology and Hepatology, University Medical Center Leiden, Leiden, The Netherlands

## Abstract

**Background:**

A decrease in the need for liver transplantations (LTX) in Primary Biliary Cirrhosis (PBC), possibly related to treatment with ursodeoxycholic acid (UDCA), has been reported in the USA and UK. The aim of this study was to assess LTX requirements in PBC over the past 20 years in the Netherlands.

**Methods:**

Analysis of PBC transplant data of the Dutch Organ Transplant Registry during the period 1988-2008, including both absolute and proportional numbers. The indication for LTX was categorized as liver failure, hepatocellular carcinoma or poor quality of life (severe fatigue or pruritus). Data were analysed for two decades: 1.1.1988-31.12.1997 (1^st^) and 1.1.1998-31.12.2007 (2^nd^). The severity of disease was quantified using MELD scores. To fit lines which show trends over time we applied a linear regression model.

**Results:**

A total of 110 patients (87% women) was placed on the waiting list. 105 patients were transplanted (1^st^: 61, 2^nd^: 44), 5 (5%) died while listed. The absolute annual number of LTX for PBC slightly decreased during the 20 year period, the proportional number decreased significantly. At the time of LTX the mean age was 53.6 yrs. (1^st^: 53.4, 2^nd^: 53.8), the mean MELD score 13.9 (1^st^:14.5, 2^nd^:13.0). The median interval from diagnosis to LTX was 90.5 months (1^st^:86.5, 2^nd^: 93.5). 69% of patients was treated with UDCA (1^st ^38%, 2^nd ^82%).

**Conclusions:**

Over the past 20 years the absolute number of LTX for PBC in the Netherlands showed a tendency to decrease whereas the proportional decrease was significant. There was a trend over time toward earlier transplantation.

## Background

In the past Primary Biliary Cirrhosis (PBC) was one of the main indications for liver transplantation (LTX), whereas nowadays, the proportion of patients receiving a transplant for PBC has decreased to around 10% [1-3]. This could possibly be due to an increase in the number of patients transplanted for other indications, to a decrease in the need for transplantation in PBC or to a combination of these factors. A decrease in need for LTX in PBC could correspond with the finding of several recent long-term cohort studies suggesting improved transplantation-free survival for ursodeoxycholic acid (UDCA) treated PBC patients, particularly for those with a favorable biochemical response upon treatment [4-6]. The impression exists that in addition to a proportional decrease in the need for LTX the absolute number of patients with PBC receiving transplantation over time is gradually falling. However, detailed reports on time trends in the absolute number of LTX for PBC are sparse [1,2]. We aimed to describe transplant patterns for PBC and changes over the past 20 years in the Netherlands.

## Methods

We performed a retrospective analysis of all patients who underwent LTX in the Netherlands in the period 01.01.1988 until 31.12.2007. Only first liver transplantations were included for analysis. Patients who were listed and/or transplanted during this period were identified using the Dutch Organ Transplant Registry (NOTR). Name, date of birth, indication and date of LTX were obtained from this database. Additional individual data were extracted from medical records in the 3 liver transplant centers in the Netherlands: the University Medical Centers in Groningen, Leiden and Rotterdam. NOTR and the liver transplantation centers gave permission to publish these data.

Laboratory data collected included alkaline phosphatase (ALP), aspartate aminotransferase (AST), alanine aminotransferase (ALT), bilirubin, albumin and creatinin levels, and platelet count, prothrombin time and INR, at the time of PBC diagnosis, at the time of placement on the waiting list and at the time of surgery. Most laboratory parameters were reported as the ratio of the test result to the upper or lower limit of normal (ULN, LLN) for the laboratory performing the test.

### Definitions and statistical analysis

A definite diagnosis of PBC was made in the presence of 2 major plus 2 minor criteria or 1 major plus 4 minor criteria. Major criteria were AMA titer ≥1:20 and compatible liver biopsy, minor criteria were pruritus, jaundice, ALP ≥2-fold the upper limit of normal, serum IgM >2.8 g/l and a positive Schirmer test (test to assess tear production) [7].

We divided the time span into the decades 1.1.1988-31.12.1997 and 1.1.1998-31.12.2007. Based on serum bilirubin, INR and creatinin the UNOS MELD score (Model for End-Stage Liver Disease, http://www.mayoclinic.org/meld/mayomodel6.html) was calculated. This model is widely used to determine prognosis in patients with liver disease [8,9]. In the Netherlands the MELD score is used for stratifying patients and allocating organs since December 2006. In addition, the disease was classified based on serum albumin and bilirubin levels, resulting in classes of early (both parameters normal), moderately advanced (one parameter normal, one abnormal) and advanced (both parameters abnormal) PBC [5]. Particularly for patients transplanted in the first decade reliable data on edema were not uniformly available, precluding calculation of the Mayo Risk Score [10].

Serum bilirubin and/or albumin concentrations were not available for 28 patients at the time of diagnosis but were complete at the time of placement on the waiting list and at the time of surgery. At the time of diagnosis the MELD score could be calculated for 40 patients. However, data were available for all patients at the time of listing and transplantation.

The indications for liver transplantation were divided into three subgroups: 1. liver failure including treatment-resistant ascites and spontaneous bacterial peritonitis, recurrent variceal bleeding, progressive muscle wasting and encephalopathy, 2. hepatocellular carcinoma and 3. poor quality of life due to intractable pruritus or severe chronic fatigue.

UDCA-treatment was classified as "yes" if the patient had used 13-15 mg/kg/day UDCA for at least 2 years, as "no" if UDCA was never used or for a period less than 2 years.

Linear regression models were applied to fit lines which show trends over time for the number of LTX, severity of disease and age at the time of LTX. A P value of less than .05 was considered significant.

## Results

### Patients

Baseline patient characteristics are summarized in Table 1.

**Table 1:** Details of patients at the time of PBC diagnosis, placement on the waiting list and transplantation.

	PBC diagnosis		Waiting list		Transplantation	
	
	1st decade	2nd decade	1st decade	2nd decade	1st decade	2nd decade
	1988-1997	1998-2008	1988-1997	1998-2008	1988-1997	1998-2008
Age (mean ± SD)	45.4 ± 7.2	45.4 ± 7.6	53.1 ± 7.6	53.0 ± 7.1	53.4 ± 7.6	53.8 ± 7.2
Female n (%)	56 (92)	36 (81)	56 (92)	36 (81)	56 (92)	36 (81)
UDCA n (%)	23 (38) started	36 (82) started	23 (38)	36 (82)	23 (38)	36 (82)

Bilirubin (ULN)	1.4(0.2-16.1)*)	1.0(0.4-6.6)*)	6.3(0.5-37.7)*)	3.6(0.5-29.6)*)	7.4 (0.4-37.7)	6.4 (0.5-43.1)
Albumin (LLN)	1.1 (0.7-2.6)	1.1 (0.8-2.0)	0.8 (0.5-6.5)	0.9 (0.6-1.2)	0.8 (0.5-6.5)	0.9 (0.3-1.3)
ALP (ULN)	5.0(1.1-19.4)*)	4.3(1.1-9.1)*)	4.5 (0.4-14.5)	2.8 (0.4-16.2)	4.1(0.4-12.6)#)	2.3(0.2-7.9)#)
AST (ULN)	2.4 (0.6-8.9)	2.1 (0.6-10.8)	3.3 (0.6-8.9)	2.8 (0.6-14.7)	2.9 (0.5-26.3)	3.0 (0.8-19.4)
ALT (ULN)	3.3 (0.7-9.3)	2.7 (0.9-14.3)	2.4 (0.4-9.3)	2.3 (0.6-12.8)	2.5 (0.2-14.6)	2.4 (0.4-18.2)
Creatinin (ULN)	0.6 (0.5-0.8)	0.7 (0.5-1.3)	0.7 (0.1-1.6)	0.7 (0.4-2.5)	0.7 (0.1-1.6)	0.7 (0.5-2.7)
INR	0.9(0.7-1.9)*)	0.8(0.6-1.0)*)	1.0 (0.7-2.4)	1.0 (0.7-1.7)	1.1 (0.7-2.8)	1.0 (0.7-2.2)

MELD (mean ± SD)	7.3 ± 2.7	7.0 ± 2.1	12.4 ± 5.1*)	14.5 ± 6.0*)	14.5 ± 6.0	13.0 ± 6.6

Prognostic class n (%)						
Early	11 (18)	18 (41)	**0 (0)**	**3 (7)**	**2 (3)**	**4 (9)**
Moderately advanced	26 (43)	7 (16)	**12(20)**	**11 (25)**	**8 (13)**	**11 (25)**
Advanced	11 (18)	10 (23)	**49 (80)**	**30 (68)**	**51 (84)**	**29 (66)**
Insufficient data	13 (21)	9 (20)	**0 (0)**	**0 (0)**	**0 (0)**	**0 (0)**

*) p < 0.05 **bold**) p < 0.001

Laboratory values are expressed as the median of the number of times the upper or lower limit of normal (range between brackets).

In total 105 PBC patients had a first liver transplant during the study period, eighty-eight percent was female; the mean age at the time of LTX was 53.6 years. The median time from PBC diagnosis to the moment of LTX was 90.5 months; the median time on the waiting list was 4.1 months.

### Changes in transplantation patterns over time

In the first decade (1.1.1988-31.12.1997) 61 PBC patients were transplanted compared to 44 patients in the second decade (1.1.1998-31.12.2007). In the first decade PBC was the cause of liver disease in 11.7% of individuals undergoing transplantation compared to 4.5% in the second decade.

**Figure 1:**
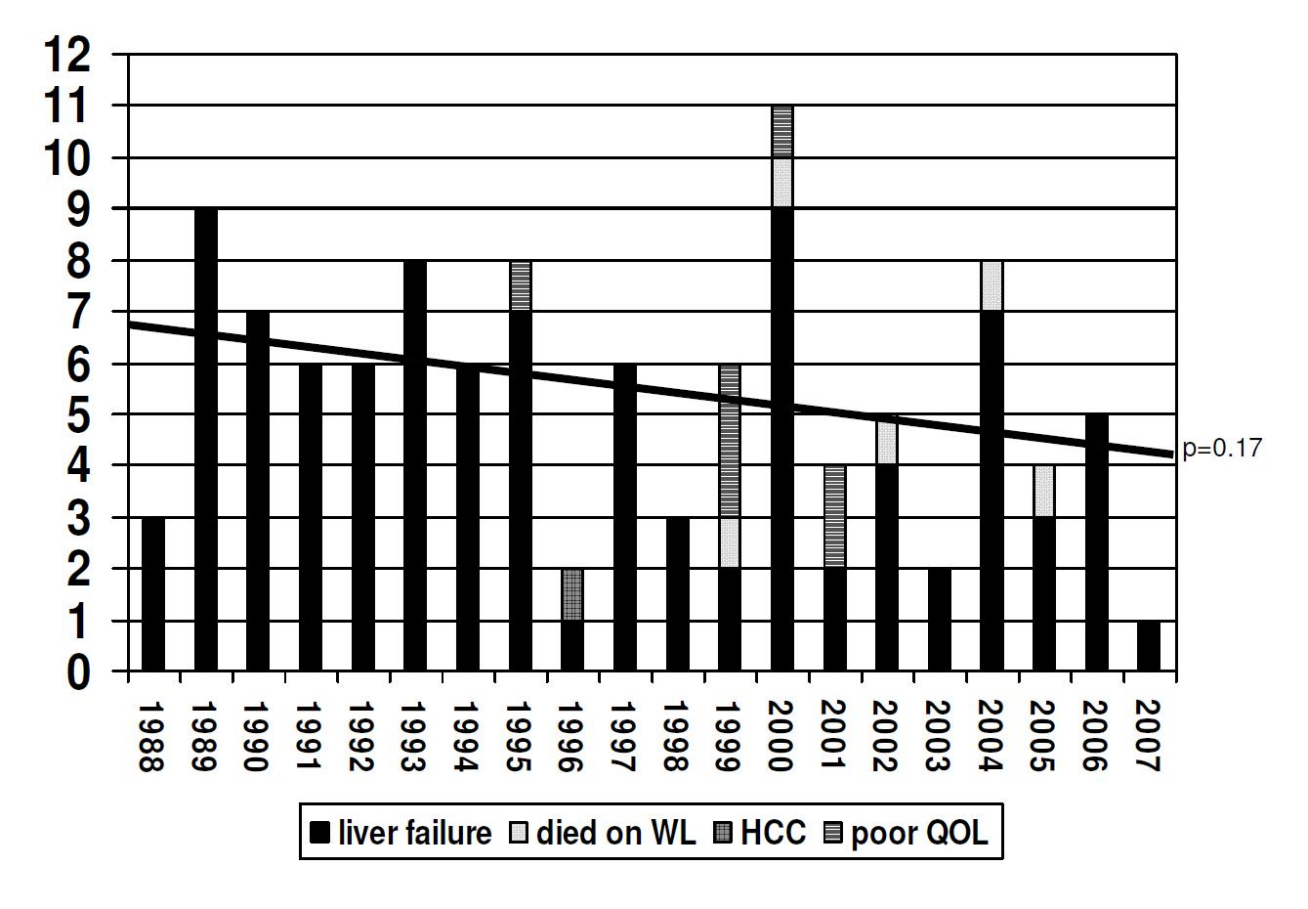
**Indications, waiting list mortality and annual absolute number of first-time liver transplantations for PBC in the Netherlands from 1988 to 2008**. The linear interpolation line indicates a decrease over time.

**Figure 2:**
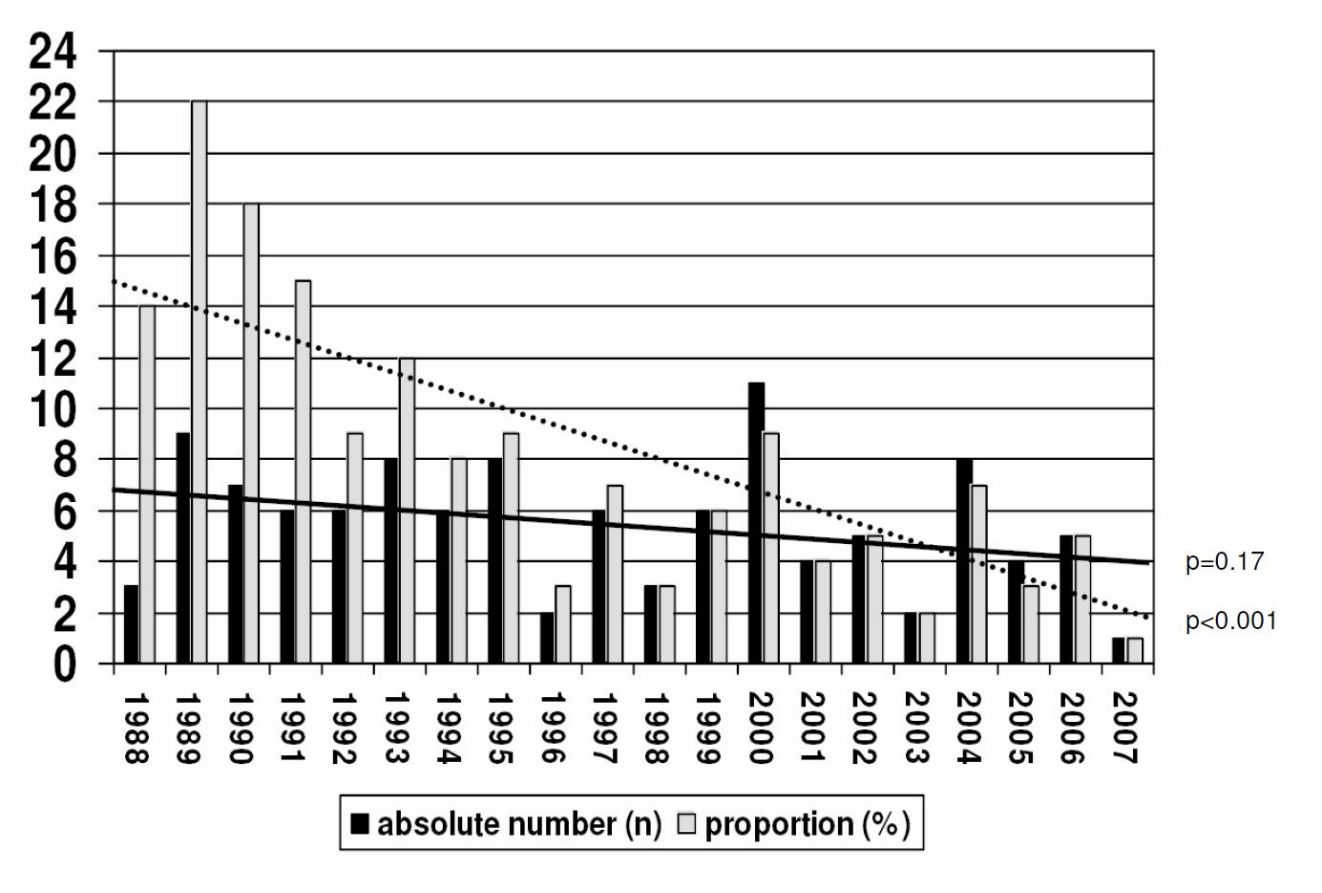
**Annual absolute number of first-time liver transplantations for PBC versus proportional number in the Netherlands from 1988 to 2008**. The linear interpolation lines indicate a decrease over time.

Figure 1 visualizes a trend to a decrease in the absolute numbers of LTX for PBC, including 5 patients who died on the waiting list (p = 0.17), the proportional decrease is significant (p < 0.001) (figure 2). As shown in table 1, the age of patients at the time of PBC diagnosis, listing and transplantation was comparable for the two decades.

Bilirubin and ALP levels were lower in the second decade both at the time of PBC diagnosis and at the time of listing and surgery. Also, the MELD scores and the proportion of patients with advanced PBC at the time of transplantation decreased over time, p = 0.14 and p = 0.03 respectively (trend-test).

The median interval from PBC diagnosis to the moment of LTX was 86.5 months in the first decade, and 93.5 months in the second decade. The median time on the waiting list was 2.3 months and 8.5 months, respectively.

### LTX & indication

The indication for LTX was liver failure in 97 (92%) cases (figure 1). 1 (1%) patient underwent liver transplantation for the presence of hepatocellular carcinoma and 7 (7%) patients for poor quality of life due to fatigue or pruritus. In the latter group, the mean age at the time of LTX was 48.3 years, the median interval between diagnosis and transplantation was 58.8 months, and the median time on the waiting list was 6.0 months. 6/7 (86%) of patients grafted for poor quality of life were transplanted early in the second decade (figure 1).

### Mortality on the waiting list

In total, 5/110 (5%) patients (4 female) died on the liver transplant waiting list. Their mean age at the time of listing was 55.4 years, the mean MELD score was 11.3 and the median time from listing to death was 4.9 months. All deaths occurred between 1998 and 2008, corresponding with 10% (5/49) waiting list mortality in that period. Causes of death were liver failure in 4 cases and heart failure in 1.

### UDCA Treatment

In the first decade 38% of patients listed for transplantation had been treated with UDCA for at least 2 years versus 82% in the second decade.

For the total group of patients the (mean) time they had been treated with UDCA increased from 21.5 months in the first decade to 71.1 months in the second decade. The mean duration of UDCA treatment for the entire period analyzed was 42.3 months.

## Discussion

The results of this study show that both the absolute and the relative number of patients who received a liver transplantation for the indication PBC has fallen during the past 20 years in the Netherlands. The severity of disease at the time of transplantation, as indicated by serum levels of bilirubin and albumin and by MELD scores, slightly decreased while the age of the patients at the time of listing and transplantation remained unchanged.

Our results are in agreement with a recent study from the USA [1]. From 1995 to 2006 the authors observed a significant decrease in the need for liver transplantation in PBC while the total number of transplantations increased and the number of transplantations for another chronic cholestatic liver disease, namely PSC, remained stable. The introduction of UDCA as standard therapy of PBC was considered the most likely explanation for the observed decline. Our data are also compatible with a single center study from Birmingham UK, reporting a decrease in the absolute and proportional number of transplantations for PBC between 1983 and 1999. The authors also noted less advanced disease at the time of transplantation for PBC over time [2]. We cannot confirm another finding of this study, namely that the age of patients at the moment of transplantation tends to increase.

A possible explanation for our findings remains uncertain. Obviously, the actual number of liver transplantations for PBC is influenced by numerous complex factors including the incidence and natural history of the disease over time, the availability of effective medical treatment, referral patterns, selection criteria and the availability of donor organs. The incidence of PBC in our country has not been defined and therefore the potential importance of changes in time cannot be addressed properly. A noticeable increased incidence of PBC has been documented in the UK while other data suggest that the incidence worldwide at least seems stable but may be increasing [11,12]. If this also applies to our country an increase or stable number of transplantations - and not a decrease - would have been more likely. It has been established that ursodeoxycholic acid has a beneficial effect in PBC and may improve prognosis, in particular in cases showing a clear biochemical response to treatment [5].

Given the almost uniform treatment of PBC with UDCA during the last 10-15 years in the Netherlands, the use of UDCA could well be a factor contributing to the observed changes. However, it is obvious that the present study was neither designed nor suitable to adequately assess the efficacy of UDCA in PBC. In the absence of data on possible changes in referral patterns for liver transplantation the significance of this factor remains unknown. With respect to selection criteria in general there was a change over time towards acceptance of older patients. However, our data show that the age at the time of listing and at the time of surgery in PBC patients in fact did not change during the 20 year study period, similar to findings in the USA [1]. Only few individuals were transplanted for other indications than liver failure, and thus changes in selection criteria are not likely to be involved in the observed trend toward less transplantation for PBC.

This was a retrospective study and the absolute number of patients was limited. We recognize that we were unable to assess all potential factors influencing the need and actual number of liver transplantations performed in the Netherlands. On the other hand we included all patients listed and/or transplanted for PBC in our country and were able to collect and analyze relevant detailed data for all cases.

## Conclusions

Our study indicates that both the absolute and the relative number of liver transplantations for PBC tended to decrease during the past two decades. The general introduction of UDCA as the standard treatment for PBC may be a factor explaining this trend over time. However, other mechanisms could well be involved given the many complex factors determining the eventual number of patients referred for, and finally undergoing, transplantation.

## Abbreviations

LTX: liver transplantation; PBC: primary biliary cirrhosis; UDCA: ursodeoxycholic acid; ALP: alkaline phosphatase; AST: aspartate aminotransferase; ALT: alanine aminotransferase; ULN: upper limit of normal; LLN: lower limit of normal; MELD: model for end-stage liver disease; PSC: primary sclerosing cholangitis; SD: standard deviation

## Competing interests

The authors declare that they have no competing interests.

## Authors' contributions

EK: study concept and design, acquisition of data, analysis and interpretation of data, drafting of the manuscript, statistical analysis. BEH: analysis and interpretation of data, statistical analysis. HM, RM, BVH and HB: acquisition of data, critical revision of the manuscript for important intellectual content. HB: study concept and design, analysis and interpretation of data, critical revision of the manuscript for important intellectual content, study supervision.

## Pre-publication history

The pre-publication history for this paper can be accessed here:

http://www.biomedcentral.com/1471-230X/10/144/prepub
